# Skeletal muscle MRI differentiates SBMA and ALS and correlates with disease severity

**DOI:** 10.1212/WNL.0000000000008009

**Published:** 2019-08-27

**Authors:** Uros Klickovic, Luca Zampedri, Christopher D.J. Sinclair, Stephen J. Wastling, Karin Trimmel, Robin S. Howard, Andrea Malaspina, Nikhil Sharma, Katie Sidle, Ahmed Emira, Sachit Shah, Tarek A. Yousry, Michael G. Hanna, Linda Greensmith, Jasper M. Morrow, John S. Thornton, Pietro Fratta

**Affiliations:** From the Neuroradiological Academic Unit (C.D.J.S., S.J.W., A.E., S.S., T.A.Y., J.S.T.), and MRC Centre for Neuromuscular Diseases (U.K., L.Z., K.T., R.S.H., N.S., K.S., M.G.H., L.G., J.M.M., P.F.), UCL Queen Square Institute of Neurology, University College London; Blizard Institute (A.M.), Queen Mary University of London, UK; and Department of Radiology (U.K.), University Hospital Tulln, Karl Landsteiner University of Health Sciences, Tulln, Austria.

## Abstract

**Objective:**

To investigate the use of muscle MRI for the differential diagnosis and as a disease progression biomarker for 2 major forms of motor neuron disorders: spinal bulbar muscular atrophy (SBMA) and amyotrophic lateral sclerosis (ALS).

**Methods:**

We applied quantitative 3-point Dixon and semiquantitative T1-weighted and short tau inversion recovery (STIR) imaging to bulbar and lower limb muscles and performed clinical and functional assessments in ALS (n = 21) and SBMA (n = 21), alongside healthy controls (n = 16). Acquired images were analyzed for the presence of fat infiltration or edema as well as specific patterns of muscle involvement. Quantitative MRI measurements were correlated with clinical measures of disease severity in ALS and SBMA.

**Results:**

Quantitative imaging revealed significant fat infiltration in bulbar (*p* < 0.001) and limb muscles in SBMA compared to controls (thigh: *p* < 0.001; calf: *p* = 0.001), identifying a characteristic pattern of muscle involvement. In ALS, semiquantitative STIR imaging detected marked hyperintensities in lower limb muscles, distinguishing ALS from SBMA and controls. Finally, MRI measurements correlated significantly with clinical scales of disease severity in both ALS and SBMA.

**Conclusions:**

Our findings show that muscle MRI differentiates between SBMA and ALS and correlates with disease severity, supporting its use as a diagnostic tool and biomarker for disease progression. This highlights the clinical utility of muscle MRI in motor neuron disorders and contributes to establish objective outcome measures, which is crucial for the development of new drugs.

Amyotrophic lateral sclerosis (ALS) and spinal bulbar muscular atrophy (SBMA, also known as Kennedy disease) are 2 major motor neuron diseases (MNDs). ALS is a rapidly progressive and fatal disorder characterized by relentless impairment of motor function following the degeneration of the upper and lower motor neurons (LMN).^1^ In SBMA LMN degeneration, caused by an expanded cytosine-adenine-guanine (CAG) repeat in the first exon of the androgen receptor (AR) gene^2^ induces progressive disabling bulbar and limb weakness at a slower rate.^3^ At disease onset, ALS and SBMA may show similar symptoms, and distinguishing the 2 disorders is of paramount interest.^4^

There are no effective disease-modifying therapies for either ALS or SBMA, and, while promising targets for prospective therapeutics have been identified,^5^ a serious limitation for clinical trials is the shortage of sensitive and reliable outcome measures for assessing disease progression.^6^

Skeletal muscle MRI can sensitively detect muscle involvement in neuromuscular diseases^7,8^ and differentiate distinct pathologic features, such as muscular fat infiltration or intramuscular edema^9^ associated with acute denervation.^10^ Muscle MRI demonstrated promise as a biomarker of disease progression in both myopathies and neuropathies.^11^

In this study, we investigated muscle MRI in SBMA and ALS by studying the lower limb and the head-neck regions, which are characteristically affected in both disorders. We report that muscle MRI can differentiate between both diseases. Moreover, we show that quantitative muscle MRI measures correlate with the clinical severity of disease, thus supporting their validity as outcome measures for quantifying disease progression in clinical trials.

## Methods

### Study design and patient recruitment

We performed a prospective cross-sectional study assessing muscular MRI of the head-neck region and lower limbs in 21 consecutive men with SBMA and 21 consecutive male and female patients with ALS who were attending the national Kennedy disease clinic and MND clinic at the National Hospital for Neurology and Neurosurgery, Queen Square, London, UK, between 2015 and 2017. Patients with genetically confirmed mutation of AR gene were included in the SBMA group. Patients eligible for the ALS group all presented with a history of at least clinically possible disease according to revised El Escorial criteria.^12^

In addition, 16 healthy controls comparable to the patient groups concerning their demographic data were recruited as a control group. Differences in sex prevalence in the ALS and SBMA groups were taken into account, where for comparisons with patients with ALS both female and male healthy volunteers were considered, whereas patients with SBMA were compared to male healthy controls only. General exclusion criteria for all participants were concomitant neuromuscular diseases and safety-related MRI contraindications.

Data from 1 patient with ALS and 1 patient with SBMA were excluded from any further analysis due to incomplete scan examination. After MRI examinations were completed, in the SBMA group data from 3 patients had to be excluded due to insufficient image quality in the head-neck region. Two patients' data were excluded for the same reason from the ALS group. In addition, in 1 healthy control, head-neck imaging could not be acquired due to inability to tolerate lying still in the MRI scanner for the required examination time.

### Standard protocol approvals, registrations, and patient consents

The study was approved by the London–Queen Square Research Ethics Committee (11/LO/1425). Written informed consent was obtained from all participants in the study.

### Data acquisition

#### Clinical and functional testing

All participants were functionally rated using the ALS Functional Rating Scale–Revised (ALSFRS-R).^13^ In patients with SBMA, functional state was additionally scored according to the SBMA Functional Rating Scale (SBMA-FRS)^14^ and supplementary measurements with the recently introduced adult myopathy assessment tool (AMAT) were performed.^15^ All participants underwent detailed assessment including medical history and an examination of the clinical and neurologic status.

### Magnetic resonance imaging

Images of the participants' head-neck, thighs, and calves were acquired at 3T (Siemens Healthineers, Erlangen, Germany). Quantitative fat-fraction maps were produced using the 3-point Dixon technique,^16^ and T1-weighted images, used for qualitative assessment, were acquired for all 3 regions. In addition, fat-suppressed T2-weighted short tau inversion recovery (STIR) images were acquired of the thighs and calves. In total, imaging of all 3 regions took approximately 35 minutes.

### MRI data analysis

#### Semiquantitative muscle MRI analysis

Muscle fat infiltration and edema, as visualized on the T1- and STIR-weighted images, respectively, were assessed and rated in consensus by 2 readers (U.K., K.T.) blinded to clinical diagnosis, after appropriate training. T1-weighted images were visually scored using the 6-point Mercuri scale^17^ (0 = normal, 1 = mild fatty streaks, 2a = early confluence, 2b = fatty infiltration 30%–60%, 3 = fatty infiltration >60%, 4 = complete fat replacement). The presence of hyperintensities in the STIR-weighted images in the thighs and calves were visually scored on a 3-point scale as proposed by Morrow^18^ (0 = none, 1 = mild, 2 = marked).

In the head-neck region the following bulbar muscles were rated on the Mercuri scale: mastication muscles comprising pterygoideus medialis (Pm); pterygoideus lateralis (Pl); temporalis (TE); masseter (MA); swallowing muscles comprising buccinator (BU); orbicularis oris (OO); digastricus (Da/p - venter anterior/venter posterior); levator veli palatini (LvP); tensor veli palatini (TvP); and intrinsic and extrinsic tongue muscles comprising genioglossus (GG). geniohyoideus (GH); hyoglossus (HG); mylohyoideus (MH).

Bilaterally, lower limb muscles were assessed on both the Mercuri and Morrow scales, and included anterior thigh compartment comprising rectus femoris (RF), vastus lateralis (VL), vastus intermedius (VI), and vastus medialis (VM); posterior thigh compartment comprising biceps femoris (long head) and biceps femoris (short head) as one muscle (BFP); semitendinosus (ST); semimembranosus (SM); medial thigh compartment comprising adductor magnus (AM), sartorius (S), and gracilis (G); anterior calf compartment comprising tibialis anterior (TA; including extensor hallucis longus); lateral calf compartment comprising peroneus longus (PL); superficial posterior calf compartment comprising gastrocnemius lateralis (LG) and gastrocnemius medialis (MG); and deep posterior calf compartment comprising the soleus (SO; including flexor digitorum longus and flexor hallucis longus) and tibialis posterior (TP).

#### Quantitative muscle MRI analysis

A single observer (U.K.) blinded to study groups outlined the muscles in the thighs, calves, and bulbar region using ITK-SNAP software.^19^ These regions were used to calculate the mean fat fraction (FF_msc_) and cross-sectional area (CSA) for each muscle and muscle compartment. In addition, a weighted overall mean muscle fat fraction (FF_all_) was calculated at each level using the following:
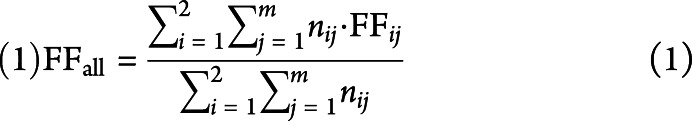
with *i*: 1 = left, 2 = right; *m* = total number of muscle regions of interest at each level.

Here *n*_*ij*_ is the number of voxels in muscle *j*. The MRI-based functional remaining muscle area (fRMA_msc_), defined as the CSA of the muscle tissue not replaced by fat, was calculated using the following:
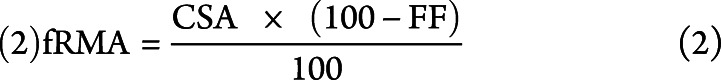


### Statistical analysis

Statistical analyses were performed with SPSS version 22 (SPSS, Armonk, NY) with an α level of 0.05. As appropriate to the distribution of data, measures are reported as mean ± SD or median and interquartile range (IQR). For intergroup comparisons, Kruskal-Wallis tests, 2-sample *t* tests, and Mann-Whitney *U* tests were applied as appropriate. Missing data were excluded from analyses. Correlations of MRI data with clinical measures were investigated with Spearman (ρ) or Pearson coefficients as appropriate for the distribution of data.

### Data availability statement

Anonymized data of this study will be shared by request from any qualified investigator.

## Results

### Participant demographics and clinical findings

The study included 2 patient groups, SBMA (n = 21) and ALS (n = 21), along with healthy controls (n = 16). For analysis exclusion criteria, see Methods. Mean age was 50.7 (SD 17) years and 54.4 (SD 14.6; *p* = 0.53) years in the SBMA and SBMA control group, respectively (table 1). Mean age was 57.3 (SD 14.8) years and 55.4 (SD 13.5; *p* = 0.69) years in the ALS and ALS control group, respectively. Height and weight did not significantly differ between the 2 patient groups and controls, except for patients with ALS having a significantly lower body mass index compared to their control group (*p* = 0.009). In both patient groups, ALSFRS-R and its lower limb (LL) subscale scores were similar (ALSFRS-R total score, SBMA: 42 [31–48], ALS: 41 [28–47]; ALSFRS-R LL subscore, SBMA: 5 [2–8], ALS: 5.5 [3–8]; ALSFRS-R bulbar subscore, SBMA: 10.5 [9–12], ALS: 12 [5–12]) and significantly reduced compared to healthy controls (SBMA vs controls; *p* < 0.001, ALS vs controls; *p* < 0.001; table 1).

**Table 1:**
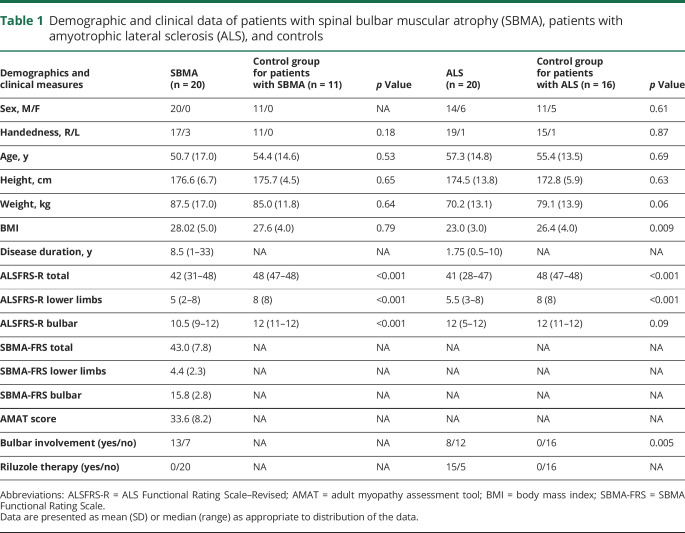
Demographic and clinical data of patients with spinal bulbar muscular atrophy (SBMA), patients with amyotrophic lateral sclerosis (ALS), and controls

### Thigh muscle fat infiltration differentiates SBMA from ALS and defines specific patterns of muscle involvement

**Figure 1:**
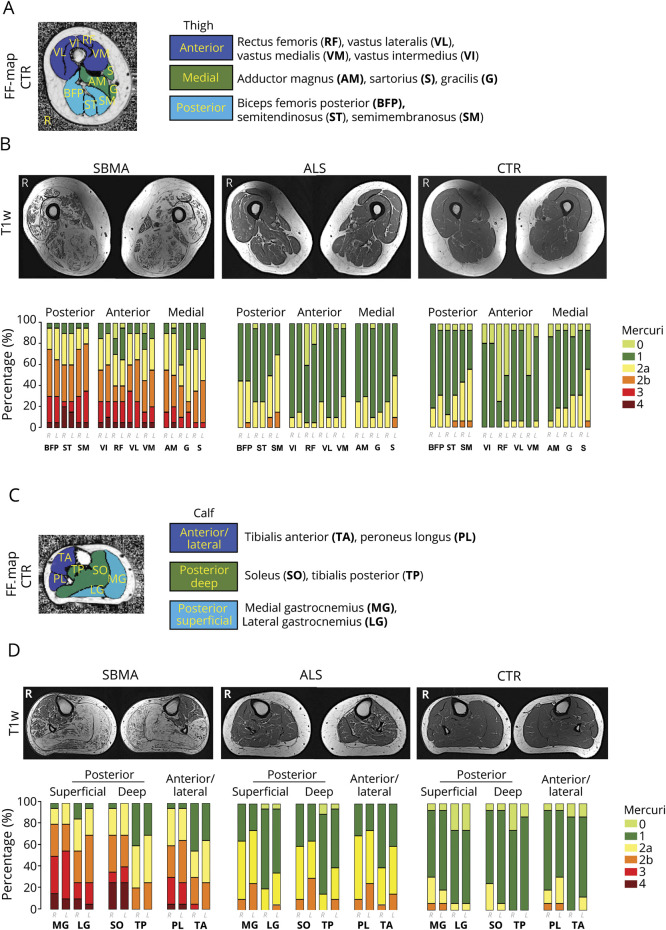
Semiquantitative muscle MRI analysis: T1-weighted imaging (A) Thigh muscle compartments and individual thigh muscles of a healthy control (CTR), superimposed on a fat fraction (FF) map. **(**B) Corresponding axial T1-weighted sample images (upper row) of the right and left thigh in spinal bulbar muscular atrophy (SBMA) (left), amyotrophic lateral sclerosis (ALS) (middle), and healthy CTR (right). Mercuri scores (lower row) are higher in the SBMA group compared to the CTR group (M-W-U = 10, *p* < 0.001). **(**C) Calf muscle compartments (CMC) and individual calf muscles of a healthy control, superimposed on a FF map. **(**D) Corresponding axial T1-weighted sample images (upper row) of the right and left calf in SBMA (left), ALS (middle), and CTR (right). Mercuri scores (lower row) are higher in both patient groups compared to their matched CTR group (SBMA vs CTR: M-W-U = 10, *p* = 0.001; ALS vs CTR: M-W-U = 130, *p* = 0.008).

To assess the pattern and the severity of muscle fat infiltration in SBMA and ALS, we acquired T1-weighted^10^ and quantitative 3-point Dixon^11^ MRI from LL muscles. We rated the T1-weighted images according to the Mercuri scale.^17^ While whole-thigh SBMA Mercuri scores were significantly increased compared to controls (median 2, IQR 1 vs median 1, IQR 0; M-W-U = 10, *p* < 0.001), there was no difference in ALS compared to controls (median 1, IQR 0 vs median 1, IQR 0; M-W-U = 130, *p* = 0.35; figure 1B). We identified a previously unrecognized pattern of muscle involvement in the SBMA patient group, affecting both anterior and posterior thigh muscle compartments (TMC) with relative sparing of the medial TMC. Specifically, the VL and the SM were the most severely affected muscles in the anterior and posterior TMC, while in the medial TMC no muscle showed severe involvement (Mercuri grade 4). In contrast to SBMA, no patient with ALS showed severe muscle fat involvement by scoring Mercuri grade 3 or 4 in any of the TMC (figure 1B).

**Figure 2:**
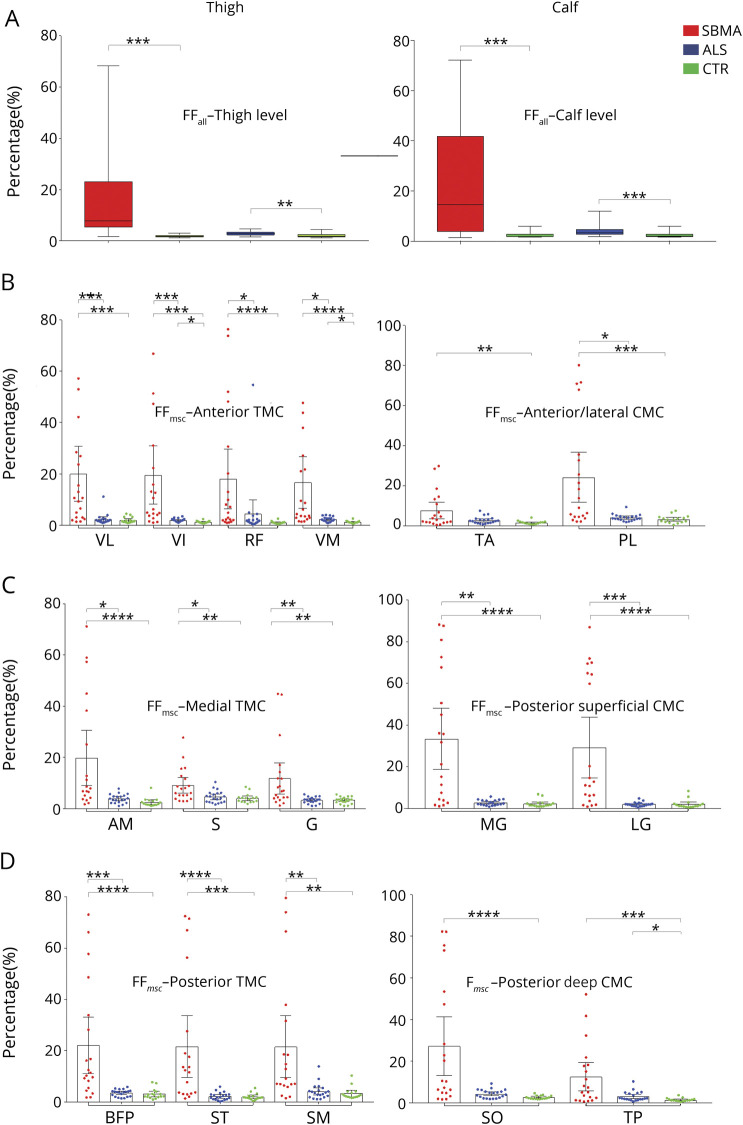
Quantitative muscle imaging: Thigh and calf muscle compartments (A) Overall muscle fat fraction percentage (FF_all_) at thigh level (left) and calf level (right) in spinal bulbar muscular atrophy (SBMA), amyotrophic lateral sclerosis (ALS), and controls (CTR). Boxes represent median and confidence interval (CI), whiskers show range. FF_all_ is significantly increased at thigh and calf level in SBMA and ALS groups compared to their age-matched controls (thigh: SBMA vs CTR: M-W-U = 12, *p* < 0.001; ALS vs CTR: M-W-U = 75, *p* = 0.006; calf: SBMA vs CTR: M-W-U = 31, *p* = 0.001; ALS vs CTR: M-W-U = 69, *p* = 0.003). **(**B) Muscle-specific fat fraction (FF_msc_) of right anterior thigh muscle compartments (TMC) (left) and right anterior/lateral calf muscle compartments (CMC) (right). **(**C) FF_msc_ of right medial TMC (left) and right posterior superficial CMC (right). **(**D) FF_msc_ of right posterior TMC (left) and right posterior deep CMC (right). Data are shown as dot plots with boxes representing mean and whiskers representing 95% CI. Asterisks indicate *p* values of post hoc pairwise comparisons between study groups of Kruskal-Wallis test results for each right thigh muscle. *0.05; **0.005; ***0.0005; ****<0.0001. AM = adductor magnus; BFP = biceps femoris posterior; G = gracilis; LG = gastrocnemius lateralis; MG = gastrocnemius medialis; PL = peroneus longus; RF = rectus femoris; S = sartorius; SM = semimembranosus; SO = soleus; ST = semitendinosus; TA = tibialis anterior; TP = tibialis posterior; VI = vastus intermedius; VL = vastus lateralis; VM = vastus medialis.

FF_all_ at mid-thigh level was significantly higher in both patient groups compared to the matched control group (SBMA vs controls: median 7.9%, IQR 17.93% vs median 1.67%, IQR 0.85%; M-W-U = 12; *p* < 0.001; ALS vs controls: median 2.79%, IQR 1.13% vs median 1.79%, IQR 0.98%; M-W-U = 75; *p* = 0.006; figure 2A). FF_msc_ confirmed the pattern identified in the semiquantitative assessments: in patients with SBMA, the highest FF_msc_ was observed in the posterior TMC (BFP, followed by ST and SM; figure 2D) and in the anterior TMC (VL, followed by VI and RF; figure 2B); the medial TMC was relatively spared, with the lowest FF_msc_ in patients with SBMA (figure 2C). Despite a significantly higher overall FF_all_ compared to their matched controls, no specific pattern of fat infiltration was observed on single muscle analysis in patients with ALS. However, there was significant atrophy of thigh muscles in patients with ALS compared to controls (table 2).

**Table 2:**
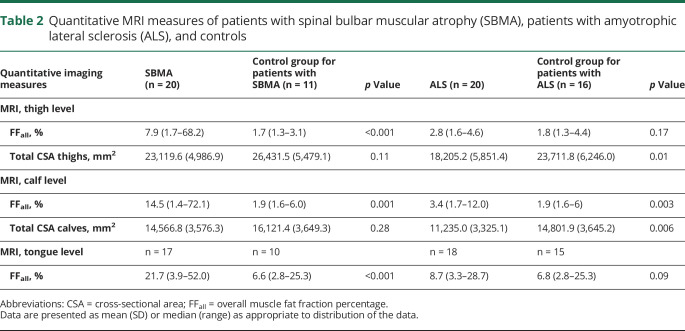
Quantitative MRI measures of patients with spinal bulbar muscular atrophy (SBMA), patients with amyotrophic lateral sclerosis (ALS), and controls

Overall, widespread intramuscular fat accumulation was observed in patients with SBMA, occurring with a preferential involvement of muscles in the anterior and posterior TMC.

### Calf muscle fat infiltration differentiates SBMA from ALS and defines specific patterns of muscle involvement

Mercuri scores for calf T1-weighted images were significantly altered in both patients with SBMA and patients with ALS compared to their matched controls (SBMA vs controls: median 3, IQR 1.88 vs median 1, IQR 0; M-W-U = 10, *p* = 0.001; ALS vs controls: median 1.5, IQR 1 vs median 1, IQR 0; M-W-U = 130, *p* = 0.008; figure 1D). Patients with SBMA showed a previously nondescribed pattern of muscle involvement with relative sparing specifically of the TA and TP muscles and predominant involvement of the superficial and deep posterior calf muscle compartments (CMC), with more than 50% of patients scoring Mercuri grade 3 or 4 in MG, SO, and LG muscles. Conversely, none of the patients with ALS or healthy controls had Mercuri grade 3 or 4 in any of the CMC. However, more than 70% of patients with ALS showed moderate fatty degeneration (Mercuri grade 2a or 2b) in the lateral CMC, affecting mostly the PL.

Quantitatively, FF_all_ were higher in both patient groups compared to controls, albeit with much lower levels in ALS (SBMA vs controls: median 14.54%, IQR 38.17% vs median 1.87%, IQR 1.23%; M-W-U = 31; *p* = 0.001; ALS vs controls: median 3.34%, IQR 2.07% vs median 1.92%, IQR 1.08%; M-W-U = 69; *p* = 0.003; figure 2A). Analysis of the FF_msc_ confirmed the semiquantitative findings of predominant posterior CMC affection and sparing of TA and TP in SBMA patient group. At calf level, the highest FF_msc_ were found in the superficial posterior CMC. Although FF_msc_ of specific calf muscles were generally lower in ALS than SBMA, in keeping with semiquantitative findings, the lateral CMC showed the highest FF_msc_ within patients with ALS (figure 2B). Compared to controls, significant atrophy of calf muscles in patients with ALS was observed (table 2).

Overall, widespread intramuscular fat accumulation, occurring with a specific pattern relatively sparing the TA and TP muscles and more severely affecting the posterior compartment muscles, was observed in the patients with SBMA. ALS also showed changes, albeit at lesser levels, and with a different pattern, the lateral compartment being the most affected.

### Fat-suppressed STIR images show specific changes in both SBMA and ALS

As the rapid course of ALS may contribute to the modest level of fat infiltration in this condition, we investigated whether semiquantitative fat-suppressed STIR sequences would reveal more identifiable changes deriving from quickly developing muscle denervation. Marked STIR hyperintensities were observed in almost all TMC and CMC in both patient groups compared to their age-matched controls.

**Figure 3:**
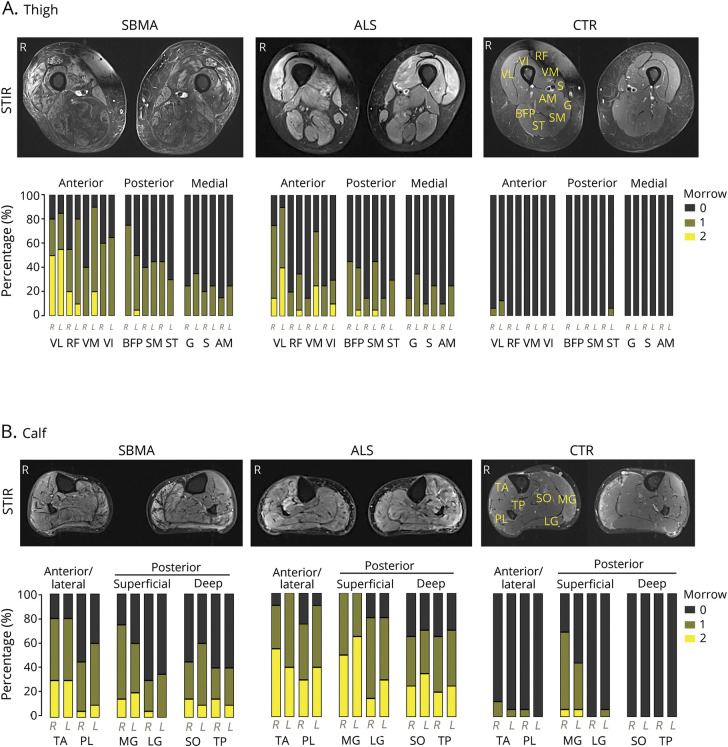
Semiquantitative muscle MRI analysis: Short tau inversion recovery (STIR) imaging (A) Corresponding sample STIR axial images (upper row) of the right and left thigh in spinal bulbar muscular atrophy (SBMA) (left), amyotrophic lateral sclerosis (ALS) (middle), and controls (CTR) (right). Proportion of Morrow scores (lower row) of thigh muscle compartments in all study groups. STIR hyperintensities were observed in both patient groups. No marked signal abnormalities were observed in healthy controls. (B) Corresponding sample STIR axial images (upper row) of the right and left calf in SBMA (left), ALS (middle), and CTR (right). Proportion of Morrow scores (lower row) of calf muscle compartments in all study groups. STIR hyperintensities were observed in both patient groups. Apart from MG, no STIR hyperintensities were observed in controls. AM = adductor magnus; BFP = biceps femoris posterior; G = gracilis; LG = gastrocnemius lateralis; MG = gastrocnemius medialis; PL = peroneus longus; RF = rectus femoris; S = sartorius; SM = semimembranosus; SO = soleus; ST = semitendinosus; TA = tibialis anterior; TP = tibialis posterior; VI = vastus intermedius; VL = vastus lateralis; VM = vastus medialis.

Using the rating scale proposed by Morrow et al.*,*^18^ marked muscle tissue hyperintensities (Morrow grade 2) were detected in the anterior and posterior TMC of patients with SBMA (figure 3A); in the calves of patients with SBMA, STIR abnormalities were observed in all muscle compartments (figure 3B). In the ALS patient group, the anterior TMC was the most altered, although signal abnormalities were detected in every CMC. In healthy controls, no marked hyperintensities were observed in any of the TMC, while in the calf, we noticed STIR signal hyperintensities of MG, previously described as “central stripe,” ^10^ which corresponds to the muscle end‐plate region of this muscle (figure 3B).

Overall, differently from T1-weighted sequences, STIR sequences detect changes in both conditions, with alterations in the calves being more marked in ALS.

### Muscle MRI detects widespread muscle changes at bulbar level in SBMA

Although bulbar muscles are affected in both SBMA and ALS, these have rarely been included in previous muscle MRI studies.^20,21^ We therefore assessed fatty changes of bulbar muscles using both T1-weighted and 3-point Dixon sequences.

**Figure 4:**
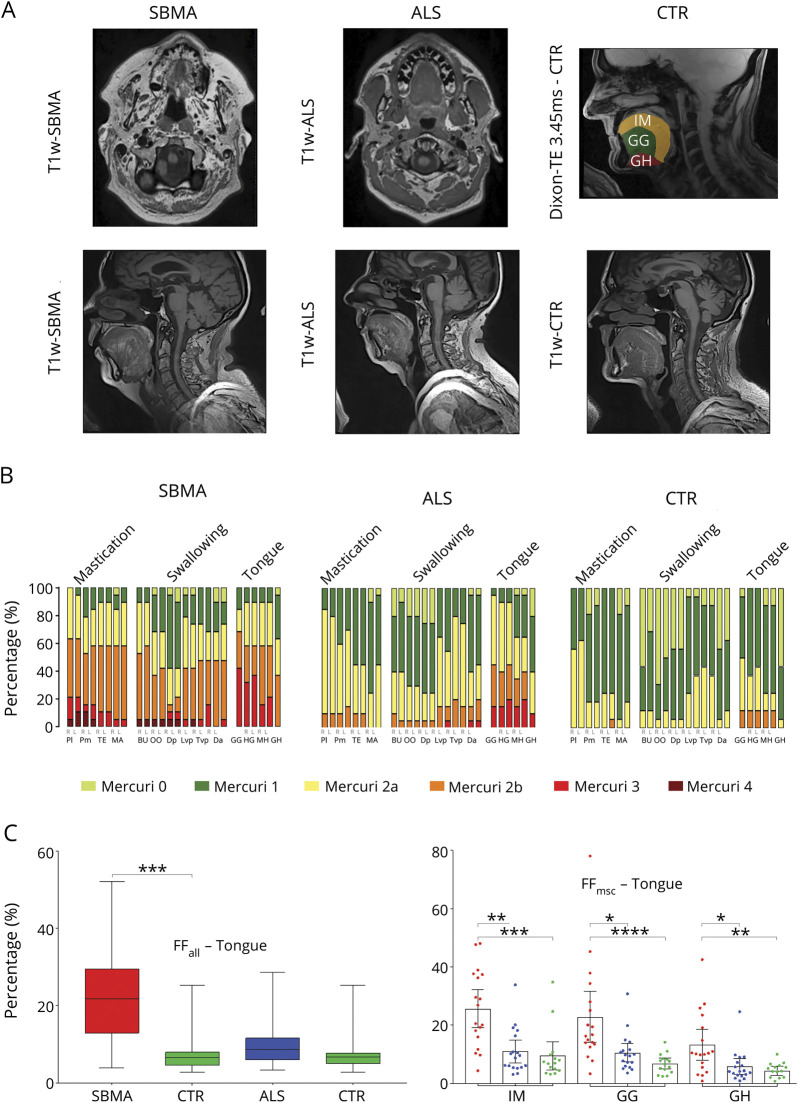
Semiquantitative and quantitative muscle MRI analysis: Head and neck region (A) T1-weighted sample images of head and neck muscles of spinal bulbar muscular atrophy (SBMA) (upper row left) and amyotrophic lateral sclerosis (ALS) (upper row middle). Regions of interest of intrinsic and extrinsic tongue muscles derived from the unprocessed shortest echo time (TE)–Dixon sequence (TE = 3.45 ms) of healthy control (CTR) (upper row right). Sagittal T1-weighted sample images of tongue muscles of SBMA (lower row left), ALS (lower row middle), and CTR (lower row right). (B) Proportion of Mercuri scores of bulbar muscles in all study groups. Mercuri scores of bulbar muscles are higher in the SBMA group and ALS group compared to their matched CTR group (SBMA vs CTR: M-W-U = 21, *p* < 0.001; ALS vs CTR: M-W-U = 90, *p* = 0.03). (C) Overall muscle fat fraction percentage (FF_all_) of tongue muscles in SBMA, ALS, and CTR (left). Boxes represent median and confidence interval; whiskers show range. FF_all_ is significantly higher in SBMA compared to controls (M-W-U = 18, *p* < 0.001). Muscle-specific fat fraction (FF_msc_) of intrinsic (IM) and extrinsic (genioglossus [GG] and geniohyoideus [GH]) tongue muscles in SBMA, ALS, and CTR (right). Asterisks indicate *p* values of post hoc pairwise comparisons between study groups of Kruskal-Wallis test results for each tongue muscle. *0.05; **0.005; *** 0.0005; ****<0.0001.

T1-weighted imaging showed a moderate to severe involvement (Mercuri grade 2b–4) of mastication and swallowing muscles in SBMA, while in contrast, patients with ALS showed predominantly only mild to moderate muscle fat infiltration of these muscle groups (Mercuri grade 1–2b; figure 4B). Both patient groups showed moderate to severe involvement (Mercuri 2b or higher) of intrinsic and extrinsic tongue muscles, including the GG, GH, HG, and MH. Notably, 12.5% of healthy controls also showed moderate muscle fat infiltration (Mercuri grade 2b) in extrinsic tongue muscles (figure 4B). Quantitative FF_all_ were significantly higher in patients with SBMA (median 21.69%, IQR 16.66%), compared to their matched controls (median 6.63, IQR 3.50; M-W-U = 18; *p* < 0.001; figure 4C). FF_msc_ were highest in the intrinsic tongue muscles, followed by extrinsic tongue muscles GG (median 16.56%, IQR 16%) and GH (median 10.29%, IQR 15%) in patients with SBMA. No significant difference in FF_all_ was seen between patients with ALS (median 8.68%, IQR 5.74%) and their matched controls (median 6.76%, IQR 2.84%; M-W-U = 94; *p* = 0.15; figure 4C).

Overall, fat infiltration in bulbar muscles is able to differentiate SBMA from both ALS and controls.

### Muscle MRI correlates with functional rating scales in MND

In order to test whether the MRI findings reflected disease severity, we calculated correlations between muscle MRI measures and functional measures, using established clinical rating instruments for each condition: the ALSFRS-R for patients with ALS and the SBMA-FRS and the AMAT score for SBMA.

**Figure 5:**
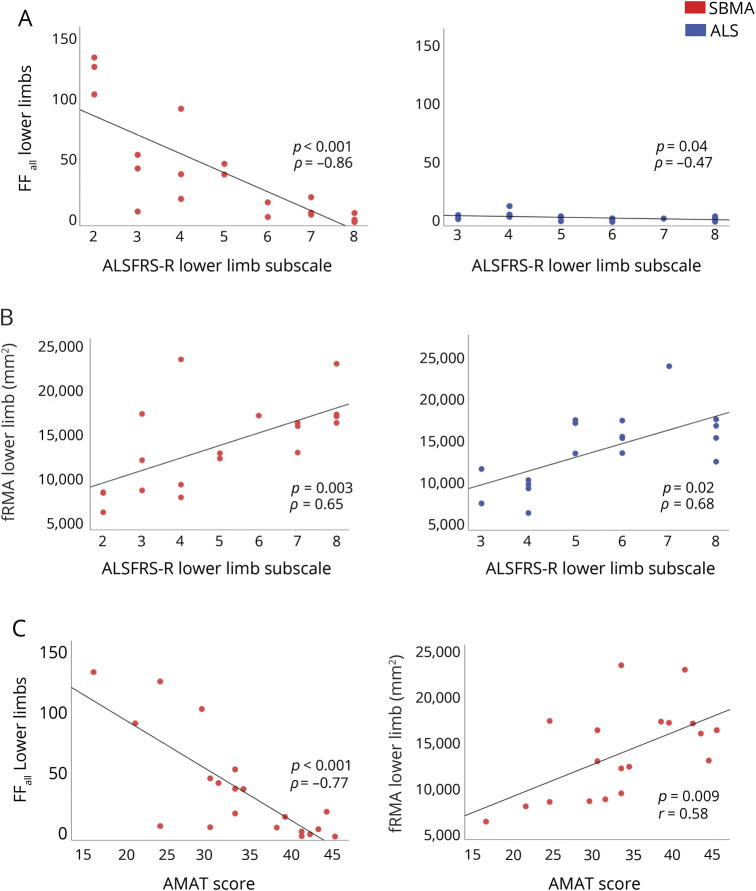
Correlation of muscle MRI measures with functional rating scales (A) Overall muscle fat fraction percentage (FF_all_) of lower limb (LL) shows a significant correlation with ALS Functional Rating Scale–Revised (ALSFRS-R) LL subscale in both patient groups (left: spinal bulbar muscular atrophy [SBMA]: ρ = −0.86, *p* < 0.001; right: amyotrophic lateral sclerosis [ALS]: ρ = −0.47, *p* = 0.04). (B) Functional remaining muscle area (fRMA) of LL shows a significant correlation with ALSFRS-R LL subscale in both patient groups (left: SBMA: ρ = 0.65, *p* = 0.003; right: ALS: ρ = 0.68, *p* = 0.02). (C) FF_all_ of LL correlates significantly with adult myopathy assessment tool (AMAT) score in patients with SBMA (left: ρ = −0.77, *p* < 0.001). Significant correlations were observed between fRMA of LL and AMAT score in patients with SBMA (right, *r* = 0.58, *p* = 0.009).

We initially asked whether the overall mean FF_all_ of combined thigh and calf level correlated with the LL subscale from ALSFRS-R and found there was a strong negative correlation between FF_all_ and disability in patients with SBMA (ρ = −0.86, *p* < 0.001) and a significant negative correlation also in patients with ALS (ρ = −0.47, *p* = 0.04; figure 5A). In SBMA, the FF_all_ strongly negatively correlated with the total AMAT score (ρ = −0.77, *p* < 0.001; figure 5C). It is important to note that FF_all_ did not correlate with age in healthy controls (ρ = −0.06, *p* = 0.8).

Since our results show that fat infiltration is not a prominent feature in ALS while active denervation changes are occurring, we next asked whether assessment of the fRMA_msc_ would take muscle atrophy into account and provide a better measure for ALS. Indeed, in patients with ALS, the fRMA_msc_ correlated strongly with ALSFRS-R LL subscales (ρ = 0.68, *p* = 0.02; figure 5B). The combined thigh and calf level fRMA_msc_ of patients with SBMA also correlated strongly with SBMA-FRS LL subscales (ρ = 0.70, *p* = 0.003) and with the AMAT score (*r* = 0.58, *p* = 0.009; figure 5C).

Overall, MRI measures correlate with functional measures in both SBMA and ALS, supporting their exploration as quantitative biomarkers of disease progression.

## Discussion

In the present study, we performed skeletal muscle MRI on cohorts of 2 major forms of adult MND: ALS and SBMA. Furthermore, for the first time, we also extended quantitative fat fraction quantitation protocols to the head and neck region, given the involvement of bulbar muscles in these diseases. In general, we show significant fat infiltration of bulbar and limb muscles in SBMA and marked STIR hyperintensities in lower limb muscles in patients with ALS. Quantitative MRI measurements strongly correlate with clinical measures of disease severity in both groups.

The results of our study have important clinical relevance; they identify a novel pattern of muscle involvement and show that quantitative and semiquantitative skeletal muscle MRI can differentiate ALS from SBMA. In addition, they support the validity of muscle MRI as a disease progression biomarker, as we show that MRI-quantified muscle FF and fRMA correlate with clinical measures of both diseases.

Muscle MRI studies in ALS^22,23^ and SBMA^24^ have mostly been limited to a small number of cases and have not included quantitative assessments.^25^ Recently, Dahlqvist et al.^26^ published results further supporting the validity of Dixon imaging in SBMA. We have studied cohorts of substantial size, using qualitative and quantitative MRI methods previously validated in other neuromuscular disease patient groups. Importantly, while a focused analysis of the lower limbs was appropriate for such conditions, we also developed reproducible protocols demonstrating the feasibility and potential value of extending MRI investigations to bulbar muscles, which are crucially involved in ALS and SBMA. While EMG is a rather restricted technique due to its invasive nature and requires complete relaxation of the tongue for appropriate recording,^27^ muscle MRI can be used to investigate specific anatomical regions at the bulbar level.

Our results suggest that different muscle changes occur in ALS and SBMA, with SBMA showing marked fat infiltration on T1-weighted MRI, while in ALS, increased edema seen on STIR images is the most prominent feature.

These findings are in line with previous observations, showing that slowly progressive neuromuscular diseases can be assessed more appropriately on T1-weighted images, while STIR imaging may detect earlier muscle pathologies such as denervation processes, before fat infiltration develops and becomes evident.^28^ Muscle denervation is a common pathologic feature in ALS,^29^ and the increase in extracellular fluid within the denervated muscle^30^ is sensitively reflected by hyperintensities on T2-weighted fat-suppressed MRI sequences such as STIR. Using whole-body MRI, Jenkins et al.^31^ recently reported higher relative T2 muscle signals in ALS compared to controls, which correlated with clinical weakness and lower motor unit number. In accord with these findings, we observed marked STIR hyperintensities in all thigh and calf muscles in both SBMA and ALS, highlighting the potential of fat-suppressed MRI sequences to reveal changes deriving from muscle denervation in the rapid course of disease progression in ALS.

Furthermore, we have identified consistent patterns of muscle involvement and sparing in SBMA. Thus, fat accumulation was observed predominantly in the posterior muscle compartment at thigh and calf level (with relative sparing of the medial thigh compartment and anterior calf compartment), and both intrinsic and extrinsic muscles at tongue level. SBMA and ALS are in differential diagnosis and approximately 13% of patients with SBMA have been reported to have previously been given a diagnosis of ALS.^32,33^ Our findings support the use of muscle MRI in the diagnostic workup of patients with prevalent LMN involvement and suspected MND. Of note, although FF for all lower limb muscles is significantly increased in SBMA, in the least affected muscles there is a strong overlap with healthy controls and patients with ALS, underlining the importance of defining muscle patterns of involvement for clinical diagnostic applications.

Importantly, we also show that the MRI-obtained fRMA_msc_ correlates with established clinical measures of disease progression in both diseases. Functional RMA takes into account both the FF_msc_ of muscles, which is crucial in SBMA, and the muscle atrophy that is typical of ALS. Indeed, while FF_all_ does significantly correlate to functional scales in ALS, the effect size is small, and longitudinal studies will be needed to assess the utility of FF measurements in ALS. The ability of MRI changes to reflect clinical involvement supports their future use as disease progression biomarkers. There is currently an intensive search for biomarkers of disease progression in MND in order to carry out more effective clinical trials.^6^ SBMA is slowly progressive and rare, and more reliable, quantitative outcome measures would reduce the duration as well as the size, and cost, of clinical trials. Survival has often been used in ALS trials,^34^ but novel sensitive outcome measures in ALS would also enable shorter trials to be undertaken, reducing costs and allowing more therapies to be tested. Muscle MRI appears to fulfil the criteria for such a biomarker, due to its reproducibility, its capability of generating quantitative measurements, and its observer independence.^35^ Furthermore, fat infiltration or denervation-related changes detected by muscle MRI may provide the ground for the development of neurochemical markers, which reflect these changes. However, further studies will be necessary to assess the sensitivity of muscle MRI to detect progressive changes in these diseases and to determine the full potential of muscle MRI as a reliable outcome measure for ALS and SBMA.

## Glossary

ALS: amyotrophic lateral sclerosis
ALSFRS-R: ALS Functional Rating Scale–Revised
AMAT: adult myopathy assessment tool
AR: androgen receptor
BFP: biceps femoris posterior
CMC: calf muscle compartments
CSA: cross-sectional area
FF_all_: overall mean muscle fat fraction
FF_msc_: mean muscle-specific fat fraction
fRMA_msc_: functional remaining muscle area
GG: genioglossus
GH: geniohyoideus
HG: hyoglossus
IQR: interquartile range
LG: gastrocnemius lateralis
LL: lower limb
LMN: lower motor neuron
MG: gastrocnemius medialis
MH: mylohyoideus
MND: motor neuron disease
PL: peroneus longus
RF: rectus femoris
SBMA: spinal bulbar muscular atrophy
SBMA-FRS: SBMA Functional Rating Scale
SM: semimembranosus
SO: soleus
ST: semitendinosus
STIR: short tau inversion recovery
TA: tibialis anterior
TMC: thigh muscle compartment
TP: tibialis posterior
VI: vastus intermedius
VL: vastus lateralis
